# Toward understanding COVID-19 pneumonia: a deep-learning-based approach for severity analysis and monitoring the disease

**DOI:** 10.1038/s41598-021-90411-3

**Published:** 2021-05-27

**Authors:** Mohammadreza Zandehshahvar, Marly van Assen, Hossein Maleki, Yashar Kiarashi, Carlo N. De Cecco, Ali Adibi

**Affiliations:** 1 School of Electrical and Computer Engineering, Georgia Institute of Technology, Atlanta, GA USA; 2 Department of Radiology and Imaging Sciences, Emory University School of Medicine, Atlanta, GA USA

**Keywords:** Viral infection, Radiography, Biomedical engineering, Computational science

## Abstract

We report a new approach using artificial intelligence (AI) to study and classify the severity of COVID-19 using 1208 chest X-rays (CXRs) of 396 COVID-19 patients obtained through the course of the disease at Emory Healthcare affiliated hospitals (Atlanta, GA, USA). Using a two-stage transfer learning technique to train a convolutional neural network (CNN), we show that the algorithm is able to classify four classes of disease severity (normal, mild, moderate, and severe) with the average Area Under the Curve (AUC) of 0.93. In addition, we show that the outputs of different layers of the CNN under dominant filters provide valuable insight about the subtle patterns in the CXRs, which can improve the accuracy in the reading of CXRs by a radiologist. Finally, we show that our approach can be used for studying the disease progression in a single patient and its influencing factors. The results suggest that our technique can form the foundation of a more concrete clinical model to predict the evolution of COVID-19 severity and the efficacy of different treatments for each patient through using CXRs and clinical data in the early stages of the disease. This use of AI to assess the severity and possibly predicting the future stages of the disease early on, will be essential in dealing with the upcoming waves of COVID-19 and optimizing resource allocation and treatment.

## Introduction

COVID-19 was declared a global health emergency by the World Health Organization in January of 2020, and governments have put unprecedented measures to halt the transmission of the virus^1^. However, the healthcare systems are still struggling with the massive influx of patients. The virus mostly emerges with mild or no symptoms in the early stages. However, it can rapidly cause pneumonia and lung opacity in patients resulting in fatality or long-term damages to the lung^2–4^. This emphasizes the importance of timely diagnosis and evaluation of the severity degree and other features of the disease to optimize resource allocation for extensive treatments such as intensive care unit (ICU).

From the beginning of the pandemic, researchers started to develop different platforms and test kits for the diagnosis of COVID-19. Prior to the availability of reverse-transcription polymerase-chain-reaction (RT-PCR) tests, chest X-rays (CXRs) and computed tomography (CT) scans were used to diagnose COVID-19^5,6^. However, there have been many cases with positive COVID-19 without any pulmonary manifestations^7^. The significant efforts and advances to foster the development of inexpensive, more accurate, faster, and easier-to-use test kits and the aforementioned reasons raise questions about using medical imaging as a tool for detecting the disease. Instead, these modalities can play a crucial role in determining the severity degree of the disease and understanding the dynamics of its development from mild to severe in different patients as well as predicting its evolution and assessing the efficacy of different treatments^2,3,8–13^, which are not feasible in RT-PCR and other laboratory test kits.

**Figure 1:**
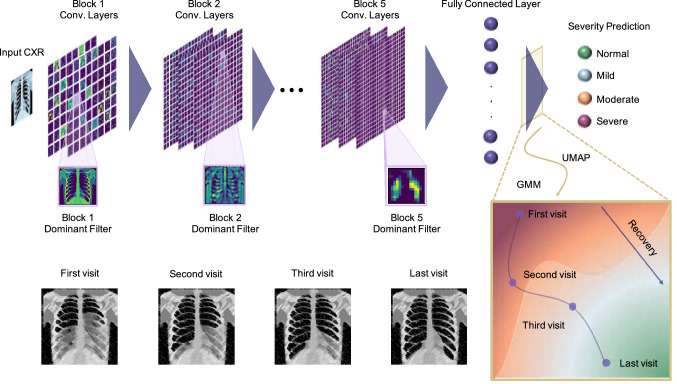
Workflow of COVID-19 severity assessment and prognosis model. The input CXR is fed into the CNN and will be processed by the network to extract the features and classify the severity degree of the disease. At each layer of the CNN, the outputs of the most dominant channels will be visualized using a pruning approach to understand the decision-making process and extracted features by the CNN. To monitor the progress of the disease, we reduce the dimensionality of the last fully connected layer using UMAP and use the Gaussian Mixture Model (GMM) to cluster the space into different severity regions. For series of input CXRs of a patient, we use the trained CNN to assess the severity degree at each visit and use the latent-space representation to visualize and monitor the progress of the disease in 2D space. Note that the X-ray images (from VectorStock) and figures are conceptual and the results for each part are presented in the following sections.

Predicting the severity degree of COVID-19 and its impacts on the lung are of great importance, enabling monitoring the progress of the disease over time and helping resource allocation in hospitals^14–16^. Several studies have shown that the severity classification and severity progression of COVID-19 are highly related to ICU admission, length of hospital stay, and optimal planning of follow-ups^6,8,10,12,13^. Despite being less sensitive than chest CT for diagnosis of COVID-19-related pneumonia, especially in the early phases of the disease, CXRs are often being utilized in the first-line assessment of patients due to their affordability and accessibility. In its recent guideline, the American College of Radiography (ACR) recommends portable CXR in ambulatory care facilities over CTs^17^. The British Medical Journal (BMJ) Best Practice recommendations for COVID-19 management also endorses the use of CXR in all patients with suspected pneumonia^18^. A retrospective study by Wong H.Y.F. et al.^4^ on 64 patients with an RT-PCR-confirmed COVID-19 diagnosis found that the most common COVID-19-related signs on CXRs were consolidation (47%) and ground-glass opacities (33%). These alterations mostly had a bilateral, peripheral, and lower zone distribution and were rarely associated with pleural effusion. In their study, 69% of patients had CXR abnormalities on the baseline X-ray, and another 11% developed CXR alterations later on in the study. As a result, CXR can be a very effective modality for monitoring the progression of the disease and its effect on the pulmonary system. However, the increasing number of patients and the large number of CXRs burden an unprecedented workload on radiologists and calls for automatic severity prediction and monitoring systems more than any time.

Artificial intelligence (AI) may be a viable solution for the automatic diagnosis and prognosis of COVID-19 and unburdening physicians and radiologists of the high workload. Recent studies in using AI for diagnosis of the disease from CXRs and CT scans shows the capabilities of these methods in providing automatic and rapid diagnosis^19–25^. However, despite the unique opportunities enabled by AI, most of the existing works focus on classification and detection of COVID-19^26–30^, rather than classifying the severity degree of the disease^31–35^ and providing intuition about its potential evolution and the decision making process, which can facilitate unprecedented knowledge discovery in COVID-19. In this paper, we demonstrate an effective AI approach for this important purpose.

While most of the reported works on CXR for diagnosis of COVID-19 use online datasets that might combine CXRs from different sources and different settings^34^, in this study, we use an authentic dataset from Emory Healthcare affiliated hospitals (Atlanta, GA, USA), which is labeled by an expert radiologist (CNDC) with 15+ years of experience. Here, we report an AI model based on training a deep convolutional neural network (CNN) for predicting the severity of COVID-19 using CXR images. We will also use interpretable models through dimensionality reduction and visualization of the outputs of different layers of the trained CNN to obtain valuable insight about the evolution of the disease as well as the decision-making process^36–39^. We show that our model can predict the severity degree of pneumonia caused by COVID-19 from CXR images with the average area under the curve (AUC) of 0.97, 0.90, 0.90, and 0.95 for normal, mild, moderate, and severe COVID-19 classes over unseen test sets. We will also visualize the most important and dominant features that the CNN extracts from CXR images at each layer of the CNN by applying a pruning algorithm based on the average percentage of zeros (APoZ)^40,41^. Finally, we leverage the uniform manifold approximation and projection (UMAP) method^42^ to form the low-dimensional manifold of the input-output relation (i.e., X-ray images to COVID-19 severity classes) to clearly monitor the disease progression.

## Results

### Emory hospital COVID-19 X-ray dataset for training and evaluation

In total, 1208 CXRs from 396 patients are used in this study. CXRs are consecutive samples from patients who had a clinically-performed positive COVID-19 RT-PCR test during the same admission as their CXR imaging at Emory-Healthcare-affiliated hospitals (Atlanta, GA, USA). An expert cardiothoracic radiologist with 15+ years of experience in reading CXRs has labeled all CXRs. These CXRs are blinded and randomized for the classification of the disease severity and the reader has had no insight into clinical data and/or outcomes. The CXRs are classified into normal, mild, moderate, and severe classes depending on the consolidation and opacity degrees (see “Methods” for more details about the labeling process). The number of images are 178, 506, 384, and 140 for the normal, mild, moderate, and severe classes, respectively (see Table 1). The dataset includes 196 males and 200 females with an average age of 63.1. We use 966 CXRs for training and keep the rest for evaluating the performance of the model (see “Methods” section for more details about the training and test data splitting and validating process over independent test sets).

**Figure 2:**
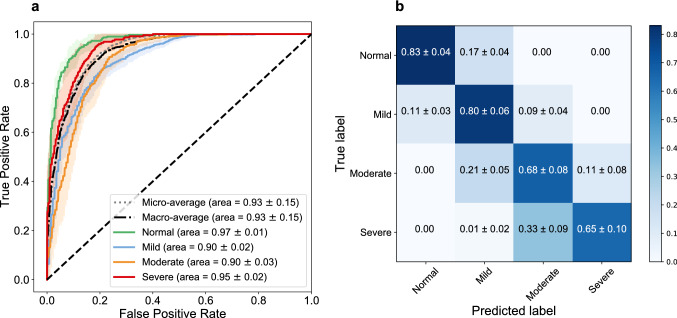
Model performance. (**a**) The receiver operating characteristic (ROC) curves for normal, mild, moderate, and severe classes and the micro- and macro-average ROCs. The solid lines show the means of ROCs over 10 independent test sets, sampled using the bootstrap method. The standard deviations for the ROCs are represented as shadows with a similar color for each class. (**b**) Confusion matrix of the model for the test datasets. The values are shown as the average performance of the model over 10 independent test sets (same as **a**) with the corresponding standard deviations. The error in non-adjacent classes (e.g., normal vs. moderate) is zero for all classes (except for severe vs. mild, which has a low error of 0.01).

**Table 1:** Patients cohort utilized in the study.

Total number of patients	396	
Total number of CXRs	1208	
CXRs class distribution	Normal	178 (14.7%)
	Mild	506 (41.9%)
	Moderate	384 (31.8%)
	Severe	140 (11.6%)
Age statistics	Min	18
	Mean	63.10
	Max	$$>90 $$
	Standard deviation	16.19
Gender distribution in CXRs	Male	640 (53%)
	Female	568 (47%)
Gender distribution in patients	Male	196 (49.5%)
	Female	200 (50.5%)

All the patients in this study are diagnosed with COVID-19 using RT-PCR test. CXRs for training and testing the model are chosen randomly from 1208 CXRs.

### Deep-learning (DL)-based model for classifying the severity of COVID-19

The CNN (shown in Fig. 1) is trained using 80% of the CXRs through a two-stage transfer-learning algorithm to assess the severity of COVID-19 for an input CXR (see “Methods” for details)^43,44^. We used class weights during the training to address the unbalanced distribution between the severity classes in the training set instead of over/under-sampling, which causes bias in the decision (see “Methods” for details). The receiver operating characteristic curve (ROC) for the average performance of the DL-based model over 10 unseen CXR test sets (sampled from the dataset using the bootstrap method^45,46^) is shown in Fig. 2a (see “Methods” section for more details regarding the validation and sampling process). The average AUC over 10 test sets for normal, mild, moderate, and severe classes are 0.97, 0.90, 0.90, and 0.95, respectively. The lower AUC for mild and moderate classes in comparison with normal and severe classes is in line with their larger overlap with other classes and the fairly subtle differences between these categories (i.e., mild and moderate) and surrounding categories (i.e., normal and severe). Based on the confusion matrix of the model (Fig. 2b), the average recall (i.e., sensitivity) for normal, mild, moderate, and severe classes is 0.83, 0.80, 0.68, and 0.65, respectively. According to Fig. 2b, the misclassification rate for non-adjacent classes (e.g., normal vs. moderate, severe vs. normal, etc.) is zero with the exception of 0.01 for the severe vs. mild case, which is negligible. The model specificity is 0.93, 0.81, 0.87, and 0.95 for the normal, mild, moderate, and severe classes, respectively. Similar to the AUC, the specificity of the model is lower for intermediate classes (i.e., mild and moderate) due to the reasons explained earlier.

**Figure 3:**
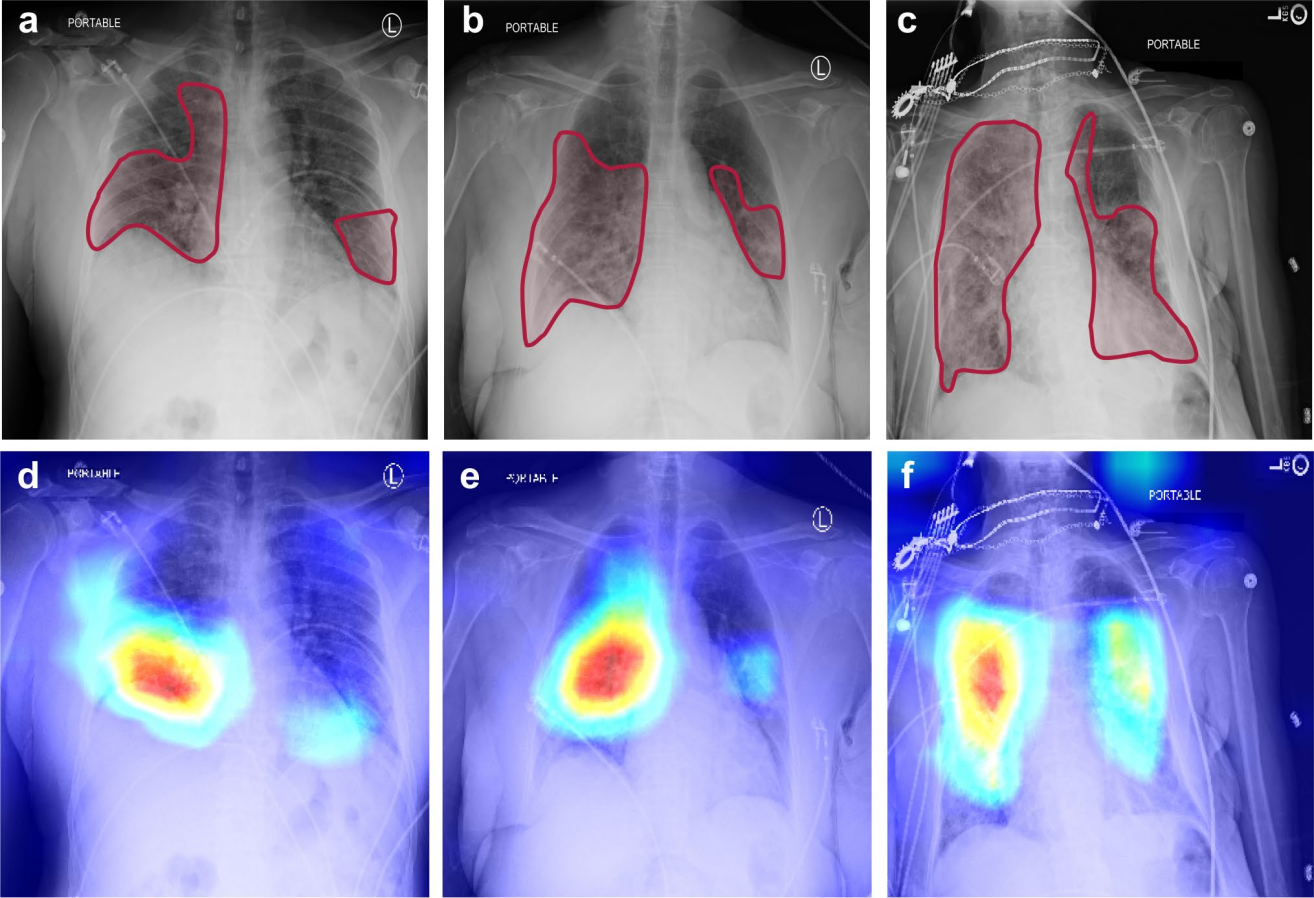
CXRs and saliency maps for three patients chosen from the unseen test dataset. The CXRs and affected areas for patients with (**a**) mild, (**b**) moderate, and (**c**) severe COVID-19 pneumonia (analyzed by our expert radiologist). (**d–f**) The corresponding saliency maps for CXRs (found by our AI approach). The true class probability for the images are 0.96, 0.67, 0.89, respectively. The saliency maps and regions of interest match well for all CXRs.

### Deep inside the layers of CNN

To assess the consistency of the results, we use the Gradient-weighted Class Activation Mapping (Grad-CAM) method for saliency map visualization of the input CXRs^37^. Figure 3a–c show the original CXRs and regions of interest (ROI) labeled by our expert radiologist for three images with mild, moderate, and severe conditions; the corresponding saliency maps obtained from the AI algorithms are shown in Fig. 3d–f. All of the images have been classified correctly by our model, and the probability of the true classes are 0.96, 0.67, and 0.89, respectively. The saliency maps demonstrate consistent activation in the regions that are affected by COVID-19 pneumonia.

**Figure 4:**
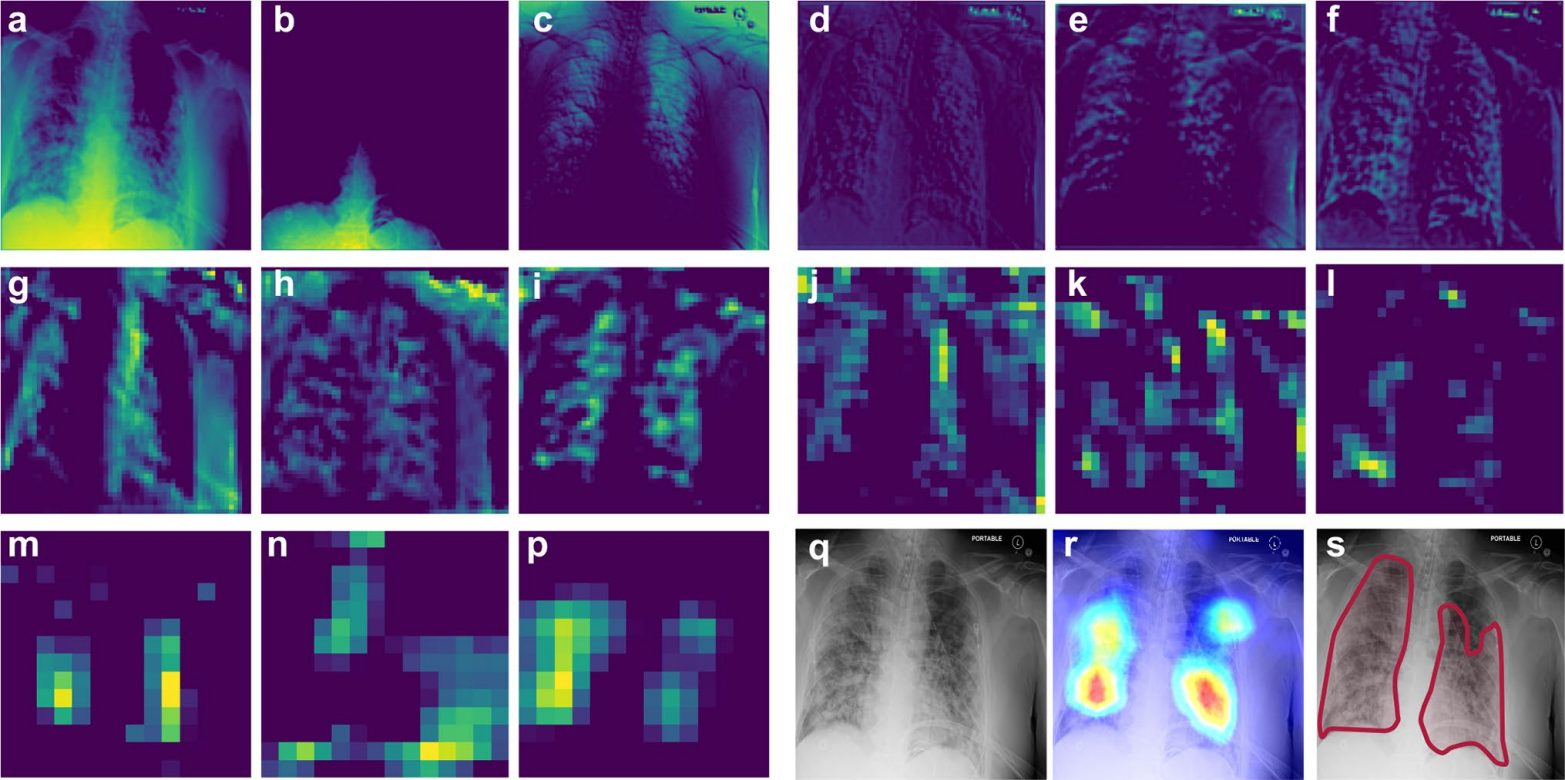
Deep inside the CNN. The representation of the dominant filters of CNN for an input CXR of a patient with severe COVID-19 pneumonia. (**a–p**) Outputs of the different CNN layers (from the first to the last convolutional layer) through the dominant filters. Each three filters correspond to one block of the CNN (see “Methods” for details). (**q**) The original input CXR for the patient. (**r**) Saliency map of the input CXR. (**s**) Affected regions labeled by our expert radiologist.

To understand the decision-making process in the CNN and visualize the extracted features, we use a pruning algorithm based on APoZ method to find the most dominant filters in each convolutional layer of the CNN. The outputs of the corresponding CNN layers under these dominant filters are shown in Fig. 4a–p for an input CXR of a patient with severe COVID-19 pneumonia. Comparing the filters to our expert radiologist’s perspective of the traditional CXRs shows that each filter highlights specific relevant parts of the CXRs similarly to the radiologist’s workflow examination. The filters from the first block of the CNN (Fig. 4a–c) focus more on highlighting different parts of the image (e.g., lung area, lower body part, etc.). In Fig. 4d–f, the filters highlight different heterogeneities of the images. Depending on the underlying anatomical structure, different parts of the CXR will have more homogenous intensities, such as the fat layers and the upper abdominal area. The aerated parts show different types of heterogeneity, caused by differences in air and fluid content. In these filters the lung fields and projected ribs are highlighted, which can help in understanding the COVID-19 disease, where we see an increased heterogeneity in the intensities of the lung fields, caused by pulmonary infiltrates. The deeper layers of the CNN extract more complex and difficult-to-interpret features (yet potentially more insightful) from the CXRs, and the final layers localize the ROIs in the image. It is clear that the large number of filters in our algorithm provides a larger range of important features and CXR areas that might offer new insights to radiologists about the disease.

**Figure 5:**
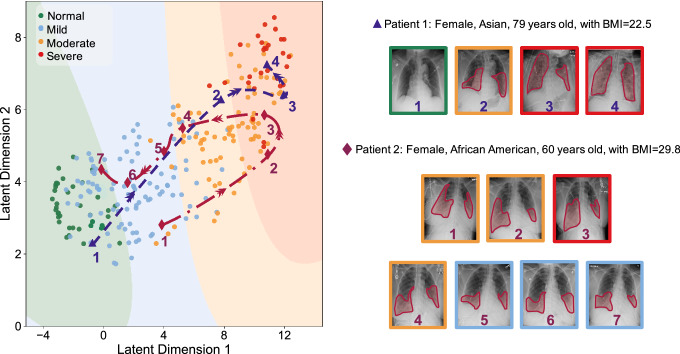
Latent space representation of the X-rays. The dimensionality of the penultimate layer of the network is reduced using UMAP, and the resulting latent space is clustered into 4 regions corresponding to different severity degrees using GMM. Green, blue, orange, and red are related to the normal, mild, moderate, and severe classes, respectively. The datapoints for each class are shown as circles with the corresponding color. The progressions of the disease over time for two patients are shown by the dashed blue (following a progression path) and red (following a recovery path after progression) paths in the latent space. Image segmentation is used as an indication of areas of interest and they are not necessarily quantitative measures for the severity (see Methods for more details about the severity assessment).

### Manifold learning for better CXR image visualization and analysis

We reduce the dimensionality of the output of the penultimate layer in the CNN in Fig. 1 using UMAP to visualize the distribution of the data in a two-dimensional (2D) space, called the latent space, and compare different COVID-19 severity classes (see Methods for details). Next, we cluster the latent space using GMM into different severity regions. Fig. 5 shows the output of the model and the regions of normal, mild, moderate, and severe classes in green, blue, orange, and red colors. The results shown in Fig. 5 support the robustness of our model in classifying non-adjacent classes, as there is no overlap between their corresponding regions in the latent space. More importantly, this approach provides a visualization tool for monitoring the progression of the disease and studying the changes in the severity degree of COVID-19 for a specific patient over time. Progressions of the disease in consecutive follow-up visits for two patients are shown in Fig. 5 with their corresponding CXRs. Patient 1 is a 79 years old Asian female with body mass index (BMI) of 22.5. As it is shown in Fig. 5, the patient has normal CXR in the first visit and the severity increases in the follow-ups and unfortunately dies. The severity path and CXRs for patient 2 (60 years old African American female with BMI of 29.8) are also shown in Fig. 5. This patient starts from mild stage and as the disease progresses, the CXR shows severe condition in the 5th follow-up and fortunately starts recovering in the next visits.

## Discussion

With the availability of the simpler, more reliable, and faster RT-PCR test kits, the strength of AI approaches must be utilized to provide early prognostication and more subtle details about the disease than just the basic detection. With pneumonia being a catastrophic feature of COVID-19, there is an urgent need for better understanding the dynamics of the progression of the adverse effects of COVID-19 on the lung. X-ray imaging can play an important role in this endeavor as the first-line imaging tool, which is an important part of the standard protocols, requiring a low radiation dose, easy to disinfect, inexpensive, and widely available, especially in low-income/rural areas and countries.

In this study, we developed an AI system to assess the severity degree of COVID-19 pneumonia using CXRs and monitor the progression. Figures 2, 3, and 5 clearly show that our algorithm can learn to assess the severity degree of COVID-19 and can be employed for monitoring the disease progression over time. Our algorithm can also provide more features than a typical CXR using the outputs of the CNN layers under dominant filters. As shown in Fig. 4, the initial layers of the CNN extract low-level features (e.g., lung areas, ribs, edges, etc.) while the final layers extract high-level and more abstract features and are not easy to interpret. Having these different views of a single CXR can help radiologists to better evaluate the severity of the disease and understand the decision-making process of the model. To the best of our knowledge, this is the first time that an interpretable model beyond saliency maps is presented in the literature for severity analysis of COVID-19. The accuracies obtained for the severity classification (Fig. 2) are good, especially knowing that the comparison is made with the labeling of only one expert radiologist. Aside from the actual classification accuracies, the separation of different severity classes in Fig. 5 clearly shows that our algorithm successfully distinguishes different classes with maximum spatial distance in the latent space between the normal and severe classes. It is also trivial why the errors in classifying mild and moderate classes are higher than those in the other two. A valuable use of the manifold-learning approach in Fig. 5 is the observation of the disease progression path for each patient. Interestingly we see that these patients take two different disease progression paths with different specifics. Patient 2, initially worsens, showing increasing signs of COVID-19-related pneumonia on sequential CXRs. However, after day 5, the patient starts recovering, showing improvement on CXRs and on day 20 is successfully discharged without re-admission. Patient 1 does not show this turning point and shows increasing disease severity on the CXRs and unfortunately dies. Understanding such turning points based on available data and relating them to the influencing factors on the disease progression are essential to create more insight into the disease trajectory to enable intelligent prediction of the disease evolution and assessment of treatment options for specific patients early on.

One of the limitations of this study is using the labels from just one radiologist for training the model. That also limits the performance and confidence of the model in the intermediate severity classes (i.e., Mild and Moderate) more than the others. To improve the performance of the model and have a better comparison with the readings of human experts, we need to gather labels from multiple radiologists for training and testing the presented approaches. Besides, having more frequent CXRs for more patients will enable the algorithm to provide valuable insight about the dynamics of the disease progression and its relation to the clinical data and treatment details over time. This can also help us to develop a more concrete model to predict at an early stage the future course of the disease for different patients and identify the patients that might need more intensive treatment form those that are more likely to recover. It can also be used to predict the best treatment approach at each stage of the disease for each patient. In addition, evaluation of the performance of the model over online benchmark datasets and multinational datasets will be needed to understand the efficacy of AI methods in clinical practice. Unfortunately, this is not a straightforward task at this stage since available online data lack, in most cases, the source of the data and related clinical features (e.g., the results of the PCR test).

It is important to note that the applicability of our AI approach for clinical data analysis is not limited to COVID-19 and can be extended for classification and severity analysis of a variety of different lung diseases, e.g., COVID-19 and different types of pneumonia^24,30^ . Once the algorithm is trained to learn the patterns of a disease through a series of training CXRs, the outputs of the different CNN layers under dominant filters can identify subtle patterns in the imaging data (as seen in Fig. 4 for CXRs) that can provide more detailed information than a single image. This can improve the accuracy of CXR reading by a radiologist. In a bigger picture, our algorithm can be adopted and extended to include other forms of imaging data for a wider range of medical diagnosis.

## Methods

### COVID-19 dataset population and labeling procedure

Patients included retrospectively for this study are selected as a consecutive sample who had a clinically performed positive COVID-19 RT-PCR test related to their CXR imaging date at Emory Healthcare affiliated hospitals (Atlanta, GA, USA). The CXRs are collected from January 1st, 2020 to May 1st, 2020, and the need for informed consent is waived by Emory University institutional review board (IRB). Both in-patients and out-patients are included. All CXRs during the entire hospital admission of the patient are collected, ranging from 1–30 images. CXRs are acquired in the posteroanterior (PA) or anteroposterior (AP) projection according to standard clinical protocols. Lateral X-rays were present for some patients, however not used for the current study. Depending on patient status, CXRs are taken with a portable X-ray unit and in a supine, erect, or semi-erect position. All CXRs have been labeled by an expert cardiothoracic radiologist with 15+ years of experience reading CXRs. All the images are blinded and randomized for the qualification of disease severity, and the reader had no insight into clinical data and/or outcomes. The CXRs are classified into normal, mild, moderate, and severe categories. This is done based on clinical experience of the reader in addition to the following guidelines:

Normal: no opacities and/or abnormalities, indicative of pneumonia, are present.
Mild: less than 50% of the lung area is affected by pneumonia-related abnormalities. Patchy (partly peripheral) opacities are present in the lower and mid lung zones.
Moderate: approximately 50$\%$ of the lung area is affected by pneumonia-related abnormalities. Opacities are present, often bilateral, in the mid and lower zones.
Severe: more than 50$\%$ of the lung area is affected by pneumonia-related abnormalities. Opacities are dense, often bilateral, and can affect all lung zones (lower, mid, and upper).

In addition to the images, basic demographics are collected such as age (at the scan time), body-mass index (BMI), and gender. All images are de-identified according to Health Insurance Portability and Accountability Act (HIPAA) and hospital-specific guidelines. Patients with an age above 90 were noted as > 90. DICOM headers are anonymized and only contain technique-related information, such as information about position of the X-ray (supine, semi-erect etc.) and whether it is a portable X-ray machine or not; all patient-related data are removed. Patients and imaging dates are coded to keep the longitudinal information between CXRs from the same patient. In addition, the date and time stamp, burned into the CXRs, are removed.

### DL-based severity classification model

To validate the performance of the model, we use a bootstrap sampling method (i.e., sampling with replacement) to create 10 sets of training and testing sets (80$\%$ for training and $20\%$ for testing in each set). We keep $10\%$ of the training set for validation and train the model on the remaining part after data augmentation. We train and test the performance of the model over each of these 10 sets independently and report the mean and standard deviation of the performances. Due to the limited number of CXRs, we use a two-stage transfer-learning approach for training the DL-based method for predicting the severity of COVID-19. We first use the pre-trained convolutional layers of the VGG-16^47^ network for the classification task over the Radiological Society of North America (RSNA) pneumonia dataset that includes 25,684 CXRs corresponding to normal, lung opacity, and other classes of lung pathologies (e.g., pulmonary edema, atelectasis, lung cancer, etc.). Then, we transfer the fine-tuned convolutional layers of the network and add fully connected layers to the transferred network, trained by our CXRs, for classifying the severity of COVID-19.

All the CXR images are resized to $224\times 224$ and preprocessed to scale the pixels between $-1$ and 1. We use random rotations, horizontal and vertical shifts, random shears, and random zooms to augment the training set for better generalization. The input of the network is an image with dimensions $224 \times 224 \times 3$ followed by 5 blocks of convolutional layers. The first block includes two convolutional layers with 64 channels and $3\times 3$ filters with same padding and rectified linear unit (ReLU) activation function, followed by a max pooling layer with stride of (2,2). The second block includes 128 channels with $3\times 3$ filters and a max pooling layer with stride of (2,2). Next, there are three convolutional layers with 256 channels and $3\times 3$ filters and a max pooling layer with stride (2,2). The fourth and fifth block each have 3 convolutional layers with 512 channels and $3\times 3$ filters and ReLU, followed by max pooling layer with stride of (2,2) and same padding. Next, the network includes a global average pooling layer, a flatten layer followed by a fully connected layer with 256 neurons and ReLU activation function. The last layer has 4 neurons with sigmoid activation function. The categorical cross entropy loss function is minimized using Adam optimizer during the training of the model. Since the dataset has unbalanced distribution over different severity classes, we used class weights during the training process to avoid high errors in the less common classes as well as the classes with higher clinical risk of misclassification (e.g., Severe). The cost for each class is selected to 1.5, 2, 2, 3 (normal, mild, moderate, and severe, respectively) to handle the unbalanced dataset.

To find the regions of interest for the CNN, we use the Grad-CAM method. The output of our severity classification model will be the saliency map of the input CXR and the class probabilities. Algorithms are implemented in Python using the Keras package and trained on a workstation with an NVIDIA RTX2080 GPU, a Core i7 CPU, and 32 GB of RAM.

### Filter representation model

To understand the role of convolutional layers in the CNN and the details of the decision-making process, we visualize the outputs of each layer of the CNN under different applied filters. We first find the most dominant filters by applying the APoZ pruning method over the training dataset. We keep 1$\%$ of the filters at each CNN layer with highest average activity (i.e., $\ell _0$-norm) over the training set and visualize the corresponding outputs of those CNN layers for any given input CXR.

### Dimensionality reduction and latent space representation model

The output of the penultimate layer (i.e., the last fully connected layer before the output layer of the CNN) is used for the latent space representation. We reduce the dimensionality of the data from 256 to 2. The number of neighbors in the UMAP algorithm are 5; the distance measure is correlation; and the minimum distance is 0.3. The UMAP is trained using the same training dataset on which the CNN is trained; none of the test samples are used during the training stage of the latent space representation model. After reducing the dimensionality to 2, we train a GMM to cluster the latent space into the regions for normal, mild, moderate, and severe COVID-19 pneumonia. The number of Gaussians (i.e. the constituents of the mixture in the GMM that model the distribution of the data) is selected as 4, and the labels are imposed as colors to specify the regions after training (Suppl. Information).

### Compliance

All the approaches and methods used the anonymized CXR data according to the guidelines of Emory University and Georgia Tech and approved by the IRB. All procedures followed HIPAA guidelines. The Emory IRB approved the collection of this data and waived the need of informed consent.

## Supplementary information

Supplementary Video 1.

Supplementary Video 2.

## Data Availability

The dataset that is used for the first stage of the transfer learning during the training is publicly available on https://www.kaggle.com/c/rsna-pneumonia-detection-challenge/data. The datasets generated during and/or analyzed (i.e., Emory COVID-19 CXR dataset) during the current study are available upon reasonable request. For more information, please contact Dr. Carlo De Cecco and Dr. Ali Adibi.
